# Progression of White Matter Lesion Volume and Health-Related Quality of Life in Patients with Symptomatic Atherosclerotic Disease: The SMART-MR Study

**DOI:** 10.4061/2011/280630

**Published:** 2011-10-16

**Authors:** Anne M. Grool, Yolanda van der Graaf, Theo D. Witkamp, Koen L. Vincken, Willem P. T. M. Mali, Mirjam I. Geerlings

**Affiliations:** ^1^Julius Center for Health Sciences and Primary Care, University Medical Center Utrecht, Stratenum 6.131, P.O. Box 85500, 3508 GA Utrecht, The Netherlands; ^2^Department of Radiology, University Medical Center Utrecht, 3508 GA Utrecht, The Netherlands; ^3^Image Sciences Institute, University Medical Center Utrecht, 3508 GA Utrecht, The Netherlands

## Abstract

*Objectives*. Mechanisms influencing the course of physical and mental functioning after an atherosclerotic event are unclear. We examined effects of white matter lesion (WML) activity on changes in functioning in patients with symptomatic atherosclerotic disease. *Methods*. In 486 patients (58 ± 9 years) of the Second Manifestations of ARTerial disease-Magnetic Resonance (SMART-MR) study, volumetric WML measurements on 1.5T MRI were performed at baseline and 3.9 ± 0.4 years followup. Functioning was assessed with the modified Short-Form 12 (SF-12) questionnaire. Associations of WML progression with changes in functioning were adjusted for age, sex, and vascular risk factors. *Results*. Physical functioning (baseline: 44, 10th–90th percentile 29–55) improved, whereas mental functioning (baseline: 51, 10th–90th percentile 32–60) declined during followup. WML progression (highest quartile versus rest) contributed to a stronger decline in mental functioning (*B* = −1.76, 95% CI −3.11 to −0.42), but did not influence changes in physical functioning. *Conclusions*. Progression of WML volume contributes to a decline in mental functioning in patients with symptomatic atherosclerotic disease.

## 1. Introduction

Ischemic heart disease and stroke are leading causes of disability and mortality worldwide [1]. As a result of improved survival and the lifelong aspect of these diseases, health-related quality of life (HRQoL), including physical and mental functioning, has become an increasingly important clinical and research outcome when evaluating burden of disease and treatment benefits. In addition, reduced physical and mental functioning not only interferes with daily living, but also increases the risk of incident ischemic vascular events and mortality [2–4]. Compared to the general population, HRQoL is substantially lower in patients with ischemic heart disease and stroke, especially in the domain of physical functioning [5–7]. A recent study indicated that HRQoL not only is lower in the acute phase of recovery from stroke, but also can decline up to five years after stroke in survivors free of recurrence or myocardial infarction [8]. Also, marked impairments in HRQoL have been observed in patients with other manifestations of atherosclerotic disease, including peripheral arterial disease [9, 10] and abdominal aortic aneurysm [11, 12].

Patients with symptomatic vascular disease frequently have atherosclerotic changes in the small vasculature in the brain, which are characterized by white matter lesions (WMLs) on magnetic resonance imaging (MRI) [13]. Although WMLs are often asymptomatic, they have been identified as a risk factor for functional decline [14], late-life depression [15, 16], and cognitive impairment [17–19]. It has been suggested that greater disease activity, characterized by an accelerated progression of WML volume, is an important underlying mechanism contributing to this elevated risk [20, 21], but longitudinal studies are still relatively scarce. Whether greater progression of WML volume is also associated with a poorer HRQoL has not been studied yet, although it could be expected that more subtle impairments in physical and mental functioning could already be present in patients with greater WML disease activity, before the development of depression or functional decline. In addition, it is unknown whether the influence of WML progression on physical and mental functioning is comparable between patients with different locations of symptomatic atherosclerotic disease.

Our first aim was to investigate the course of physical and mental functioning in patients with different manifestations of atherosclerotic disease over four years of follow-up. Second, we examined whether greater progression of WML volume contributed to poorer physical and mental functioning in these patients and whether these associations depended on the location of symptomatic atherosclerotic disease.

## 2. Materials and Methods

### 2.1. Participants

Data were used from the Second Manifestations of ARTerial disease-Magnetic Resonance (SMART-MR) study, a prospective cohort study aimed to investigate brain changes on MRI in 1309 independently living patients with symptomatic atherosclerotic disease. Details of the design and participants have been described elsewhere [13]. For the current study, data were used from 989 patients newly referred to the University Medical Center Utrecht between January 2002 and December 2005 with manifest peripheral arterial disease, coronary artery disease, cerebrovascular disease, or abdominal aortic aneurysm without MR contraindications and available data on the HRQoL questionnaire. During a 1-day visit to our medical center, an MRI of the brain, physical examination, blood, and urine sampling were performed. Risk factors, medical history, and functioning were assessed with questionnaires.

Between January 2006 and May 2009, all participants still alive were invited for follow-up measurements, including MRI of the brain, neuropsychological testing, a physical examination, blood and urine sampling, risk factors, medical history, and functioning. The SMART-MR study was approved by the ethics committee of our institution, and written informed consent was obtained from all participants.

In total, 585 of the surviving cohort (62% of *n* = 943) gave written informed consent; 346 (37%) persons refused, and 12 (1%) were lost to followup.

### 2.2. Magnetic Resonance Imaging Protocol

MR investigations were performed on a 1.5-tesla whole-body system (Gyroscan ACS-NT, Philips Medical Systems, Best, The Netherlands). The protocol consisted of a transversal T1-weighted gradient-echo sequence (repetition time (TR)/echo time (TE): 235/2 ms; flip angle, 80°), a transversal T2-weighted turbo spin-echo sequence (TR/TE: 2200/11 ms and 2200/100 ms; turbo factor 12), a transversal T2-weighted fluid attenuating inverse recovery (FLAIR) sequence (TR/TE/inversion time (TI): 6000/100/2000 ms), and a transversal inversion recovery (IR) sequence (TR/TE/TI: 2900/22/410 ms) (field of view 230 × 230 mm; matrix size, 180 × 256; slice thickness, 4.0 mm; no gap; 38 slices).

### 2.3. Brain Segmentation

We used the T1-weighted gradient echo, IR sequence, and FLAIR sequence for brain segmentation. The probabilistic segmentation technique has been described elsewhere [22, 23]. The segmentation program distinguishes cortical gray matter, white matter, sulcal and ventricular cerebrospinal fluid (CSF), and lesions. The results of the segmentation analysis were visually checked for the presence of infarcts and adapted if necessary to make a distinction between WML and infarct volumes. Total brain volume was calculated by summing the volumes of gray and white matter and, if present, the volumes of WML and infarcts. All volumes cranial to the foramen magnum were included. As a result, the total brain volume includes the cerebrum, brainstem, and cerebellum. Total intracranial volume (ICV) was calculated by summing the total brain volume and the volumes of the sulcal and ventricular CSF.

### 2.4. Infarcts and White Matter Lesions

The whole brain was visually searched for infarcts by a trained investigator and a neuroradiologist. Raters were blinded to history and diagnosis of the patient. Discrepancies in rating were reevaluated in a consensus meeting. Infarcts were defined as focal hyperintensities on T2-weighted images of at least 3 mm in diameter. Hyperintensities located in the white matter also had to be hypointense on T1-weighted and FLAIR images in order to distinguish them from WML. Dilated perivascular spaces were distinguished from infarcts on the basis of their location, form, and the absence of gliosis. The location, affected flow territory, and type were scored for every infarct.

WML volumes obtained with the segmentation program were summed to obtain total WML volume. Volumes of WML were normalized for ICV and expressed as percentage of ICV.

### 2.5. Physical and Mental Functioning

At baseline and followup, patients completed the Short Form-12 (SF-12) [24], a shortened version of the Short Form-36 (SF-36) Medical Outcomes Study Health Survey [25], to measure HRQoL at baseline and followup. The SF-12 questionnaire includes 1 or 2 items from each of the 8 health summary scales of the SF-36 [26] and enables calculation of the Physical (PCS) and Mental Component Summary scales (MCS). The SF-12 summary scales are positively scored and normalized to a general population mean of 50 with standard deviation of 10. Higher SF-12 scores indicate better HRQoL; a positive change in SF-12 scores indicates an improvement, and a negative change a deterioration in HRQoL. Because of its brevity, the SF-12 is considered advantageous over the SF-36 for large studies focusing on overall physical and mental functioning [26].

### 2.6. Severity of Atherosclerotic Disease at Baseline

In patients with peripheral arterial disease, severity of vascular disease at baseline was assessed using the Fontaine scale [27]. Stage 1 (pain-free walking distance >200 m) and stage 2 (pain-free walking distance <200 m) were defined as mild or moderate ischaemia, whereas stage 3 (rest pain) and stage 4 (ulceration or gangrene) were defined as severe ischaemia. In patients with coronary artery disease, disease severity was rated according to the number of coronary arteries with marked atherosclerosis (>70% stenosis or fractional flow reserve <0.80 or treatment of the vessel). One-vessel, two-vessel, three-vessel, left main disease with or without right coronary artery involvement was rated in all coronary artery disease patients on the basis of coronary angiography reports. Information was incomplete in some patients, and additional information was obtained from percutaneous coronary intervention or coronary artery bypass grafting reports. For patients with cerebrovascular disease, disease severity was classified with a handicap scale, the modified Rankin Scale (mRS) [28].

### 2.7. Other Variables

During the visit to the medical center, an overnight fasting venous blood sample was taken to determine glucose levels. Systolic and diastolic blood pressures (mmHg) were measured twice with a sphygmomanometer and averaged. Hypertension was defined as mean systolic blood pressure ≥160 mmHg, mean diastolic blood pressure ≥95 mmHg, or self-reported antihypertensive drug use. Diabetes mellitus was defined as fasting glucose ≥7.0 mmol/L or self-reported use of oral antidiabetic drugs or insulin. Smoking habits and alcohol intake were assessed with questionnaires. Packyears of smoking was calculated, and alcohol use was categorized into never, past, and current.

### 2.8. Study Sample

Of the 585 patients participating at followup, data on baseline or follow-up MRI variables were missing in 74 patients (no MR (*n* = 41), irretrievable MR data (*n* = 5), missing FLAIR images (*n* = 7), or artefacts (*n* = 21)). Of these, HRQoL data at followup were missing in 17 patients. Of these 494 patients, data on vascular risk factors were missing in 8 patients. This resulted in a total study sample of 486 patients.

Compared to patients who were lost to followup (*n* = 503), patients who participated at followup (*n* = 486) were significantly younger (mean 57.5 versus 59.6 years) at baseline, had less often hypertension (50% versus 57%) and diabetes mellitus (16% versus 25%), more often reported current alcohol intake (79% versus 72%), had lower WML volume (median 1.3 versus 1.7 mL), had better mental functioning (median 51.0 versus 48.3), and were less often included with peripheral arterial disease (19% versus 26%) or abdominal aortic aneurysm (5% versus 11%) (Table 1).

### 2.9. Data Analysis

First, we calculated changes in physical and mental functioning after on average 4 years of followup in the total sample and then compared changes in physical and mental functioning between different locations of symptomatic atherosclerotic disease using generalized linear models with physical and mental functioning scores at followup as the dependent variables and location of symptomatic atherosclerotic disease, age, sex, baseline physical or mental functioning, and follow-up time as independent variables.

Second, linear regression analysis was used to investigate whether greater progression of WML volume was associated with changes in physical and mental functioning. Progression of WML volume was defined as the difference in WML volume (% of ICV) between baseline and followup. We divided WML progression into quartiles, and dichotomized WML progression (highest quartile (*n* = 126) versus lower quartiles (*n* = 360) to investigate whether patients with greatest progression showed a different course of physical and mental functioning than patients with no or minimal WML progression. Analyses were first performed in the total sample, and because we expected that associations could be influenced by the type of underlying atherosclerotic disease, we repeated the analyses within strata of locations of atherosclerotic disease. In model I, associations were adjusted for age, sex, baseline physical or mental functioning and follow-up time. We additionally adjusted for smoking, alcohol use, hypertension, and diabetes mellitus in model II, because it is not clear to what extent these vascular risk factors are confounders or preceding factors in the pathway between WML volume and functioning, or both.

We repeated the analyses after excluding patients with severe atherosclerotic disease at baseline, defined as patients with coronary artery disease and three-vessel or left main disease at inclusion, patients with cerebrovascular disease and a mRS grade ≥2 at inclusion, or patients with peripheral arterial disease with Fontaine grade ≥3 at inclusion. This was done to assess to what extent the observed associations between small-vessel disease and functioning were influenced by the severity of macrovascular disease.

Further, to examine whether associations were independent of incident vascular events during followup, analyses were repeated after excluding patients who experienced a new vascular complication (nonfatal ischemic stroke or myocardial infarction) between baseline and followup. In all analyses, 95% confidence intervals are given. SPSS version 15.0 (Chicago, Ill, USA) was used to analyze our data.

## 3. Results

Baseline characteristics are summarized in Table 2. Mean age of the study population was 58 ± 9 years, and 80% was male. At baseline, median physical functioning was 44 (10–90th percentile 29–55) and mental functioning was 51 (10–90th percentile 32–60). 

**Figure 1:**
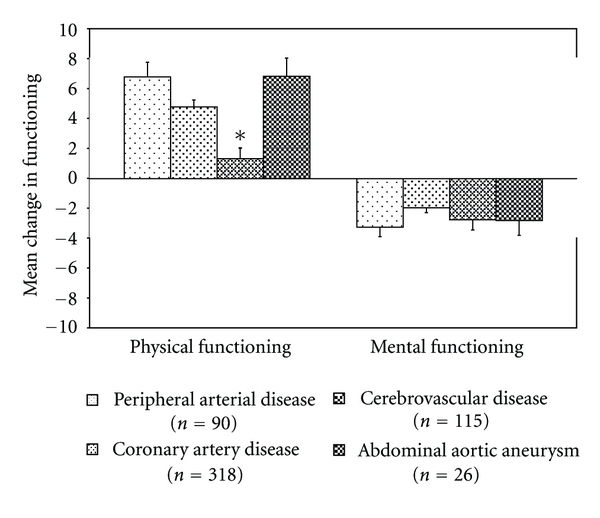
Mean changes in physical and mental functioning for different locations of symptomatic atherosclerotic disease, adjusted for age, sex, baseline functioning, and follow-up time. Significant differences, compared to other locations of symptomatic atherosclerotic disease, are indicated with an asterix.

Mean elapsed time between the vascular event and screening date was 2.1 ± 1.4 months. In the total sample, physical functioning improved (median 3.8, 10th–90th percentile −6.5 to 18.3) and mental functioning deteriorated (median −4.0, 10th–90th percentile −14.0 to 13.0) after a mean followup of 3.9 ± 0.4 years. When different locations of atherosclerotic disease were identified, physical functioning improved in all groups (Figure 1). This improvement was significantly lower in patients with cerebrovascular disease compared to patients with other locations of symptomatic atherosclerotic disease (*B* = −2.58, 95% CI −4.29 to −0.87). Mental functioning deteriorated in all groups, without any significant differences between different locations of symptomatic atherosclerotic disease (Figure 1).

### 3.1. Progression of WML Volume

**Figure 2:**
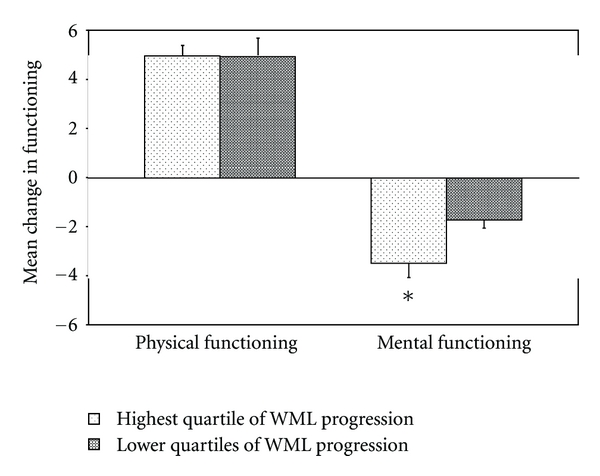
Mean changes in physical and mental functioning for patients with greatest progression of white matter lesion (WML) volume (highest quartile, >0.07% increase in WML volume as % of ICV) versus patients in the lower three quartiles of progression, adjusted for age, sex, baseline functioning, and follow-up time. Significant differences are indicated with an asterix.

**Figure 3:**
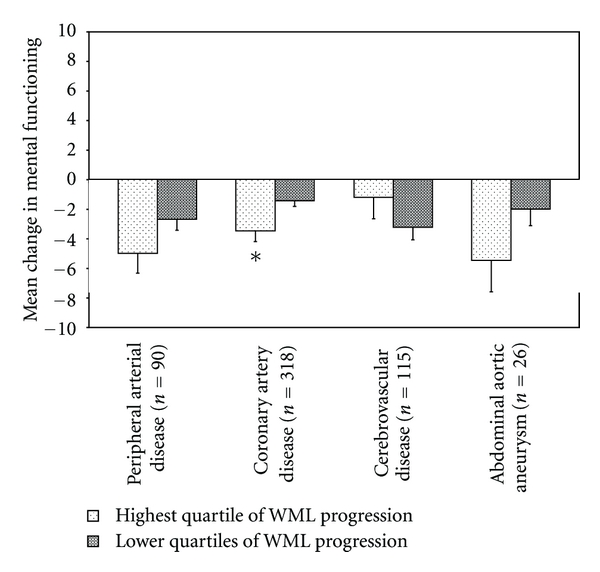
Mean changes in mental functioning for patients with greatest progression of white matter lesion (WML) volume versus patients in the lower three quartiles of progression, for different locations of symptomatic atherosclerotic disease, adjusted for age, sex, baseline functioning, and follow-up time. Significant differences are indicated with an asterix.

Patients with greatest progression of WML volume (highest quartile, >0.07% increase in WML volume as % of ICV) showed a significantly stronger deterioration in mental functioning than patients with lower WML progression (*B* = −1.76, 95% CI −3.11 to −0.42, Figure 2) in model I. Additional adjustment for vascular risk factors did not change the results (data not shown). When analyses were repeated within different strata of locations of symptomatic atherosclerotic disease, greater WML progression was associated with a stronger deterioration in mental functioning in all patients except for patients with cerebrovascular disease (Figure 3), although the deterioration was statistically significant only in patients with coronary artery disease (model I, *B* = −2.03, 95% CI −3.61 to −0.45). Additional adjustment for vascular risk factors did not change these associations.

Greater progression of WML volume was not significantly associated with changes in physical functioning at followup in model I in the total sample (*B* = −0.04, 95% CI −1.79 to 1.72, Figure 2) or within strata of patients with coronary artery disease (*B* = 0.02, 95% CI −2.26 to 2.30), peripheral arterial disease (*B* = 1.26, 95% CI −3.65 to 6.16), cerebrovascular disease (*B* = 0.00, 95% CI −3.46 to 3.46), or abdominal aortic aneurysm (*B* = −0.38, 95% CI −7.07 to 6.32).

Excluding patients with most severe symptomatic atherosclerotic disease (*n* = 46) did not materially change the results. Greater WML progression was still significantly associated with a stronger deterioration in mental functioning (model I, *B* = −1.82, 95% −3.25 to −0.38). 

Between baseline and followup, 17 patients experienced a nonfatal vascular event. Excluding these patients did not change the observed associations of greater WML progression with a stronger deterioration in mental functioning (model I, *B* = −1.83, 95% CI −3.20 to −0.46).

## 4. Discussion

In a cohort of patients with different manifestations of symptomatic atherosclerotic disease, physical functioning substantially improved in all patients after four years of followup, although the improvement was less in patients with cerebrovascular disease. Mental functioning declined in all types of symptomatic atherosclerotic disease. Greater progression of WML volume over four years of followup was associated with a stronger decline in mental functioning in all patients except for those with cerebrovascular disease.

To our knowledge, this is the first study directly investigating the influence of WML progression on the course of physical and mental functioning in patients with different manifestations of symptomatic atherosclerotic disease. A strength of this study is that by including patients with different locations of symptomatic atherosclerotic disease we could investigate whether the effect of WML progression on physical and mental functioning depended on the type of underlying vascular disease. Furthermore, volumetric WML assessment provided estimates that are more precise and less influenced by observer bias than visual rating scales [29–31] and enabled the measurement of relatively small volume changes over time. In addition, we included a large number of patients, and the extensive information available on cardiovascular risk factors and the extent of clinical and subclinical atherosclerosis made it possible to adjust for potential confounders.

A limitation of this study is that, despite the large sample size, relatively few patients had peripheral arterial disease, cerebrovascular disease, or abdominal aortic aneurysm. Although similar associations were found in patients with coronary artery disease, peripheral arterial disease and abdominal aortic aneurysm, the relatively low number of patients with locations of symptomatic atherosclerotic disease other than coronary artery disease contributed to large confidence intervals and possibly nonsignificant relations in these patients. Further, the largest impact on physical and mental functioning would be expected in patients suffering most severe atherosclerotic events. Because these patients are less likely to participate in our study, this could have contributed to a relative underestimation of the effect. Moreover, patients who participated at followup were healthier at baseline, with fewer vascular risk factors, lower WML volume, and higher mental functioning than patients lost to followup. Therefore, the changes in physical and mental functioning might have been less prominent in the total cohort. Also, because baseline mental functioning was higher in patients with complete data at followup, regression to the mean could have contributed to the observed decline in mental functioning after four years. On the other hand, the selection of relatively healthy patients could have resulted in a decreased contrast between those with greatest WML progression and those without, which could have led to an underestimation of the effect of WML progression on changes in mental functioning.

In recent years, HRQoL has become an increasingly important clinical and research outcome measure when evaluating burden of disease and treatment benefits in patients with atherosclerotic disease. Population-based studies have shown that patients with various manifestations of symptomatic atherosclerotic disease have a poorer HRQoL compared to the general population, with most pronounced effects on physical functioning [5–7, 9, 10]. It is unclear whether physical and mental functioning returns to population levels after the acute phase of recovery or whether functioning remains lower, or perhaps even further declines after the initial event.

Our data showed that physical functioning was substantially lower in patients with symptomatic atherosclerotic disease in the acute phase of recovery from an atherosclerotic event compared to previously published age-adjusted population norms [32]. In a previous population-based study, a prolonged decline in HRQoL was observed in stroke survivors free from recurrent stroke or myocardial infarction [8]. Although we found an improvement in physical functioning in our sample of patients with cerebrovascular disease after four years followup, this improvement was substantially lower compared to patients with other locations of symptomatic atherosclerotic disease. In line with our findings, another study also reported significant improvements in functioning in postoperative abdominal aortic aneurysm patients, which returned to population norms in long-term survivors [11].

In our study, mental functioning was similar to population norms in the acute phase of recovery from a vascular event, but declined during a four year follow-up period. Other studies reported an increased prevalence of mood disturbances already in the acute phase in patients hospitalized for ischemic cardiac or cerebrovascular events [33, 34]. One explanation for our findings could be that in the acute phase of an atherosclerotic event, subjective well-being is dominated by the substantial impairments in physical functioning, whereas awareness of the emotional consequences arises after recovery of physical functioning. An alternative explanation could be that the course of mental functioning in patients with symptomatic atherosclerotic disease depends on the severity of the atherosclerotic event. Relatively few patients were included with severe atherosclerotic disease in our study, which could contribute to the different findings in the course of mental functioning between our study and others.

The underlying mechanisms contributing to a lower HRQoL in patients with symptomatic atherosclerotic disease are unclear. It has been suggested that a lower HRQoL could result from direct complications of the disease or treatment of underlying vascular risk factors, or from raised awareness of the disease [35]. An alternative mechanism contributing to a lower perceived HRQoL could be the presence of co-occurring intracerebral atherosclerotic changes, characterized by WMLs on MRI. WMLs are strongly associated with the presence of common vascular risk factors, including increased age, hypertension, and diabetes mellitus [36–38]. Although the exact underlying pathophysiological mechanisms remain unclear, arteriosclerotic changes to the cerebral small vasculature, with consequent ischemia, apoptosis, and blood-brain barrier alterations, are thought to be involved in the formation and progression of WMLs [39]. Although WMLs are often asymptomatic MRI findings, increased volume and progression of WML have been previously associated with an increased risk of functional decline [14], depression [16], and cognitive impairment [18, 19]. WMLs are thought to account for the increased risks of functional decline and mood disorders by disrupting brain pathways that are involved in the regulation of physical and emotional responses [40]. Although we did not formally measure depression, our finding that increased WML activity was associated with a greater decline in mental functioning may be interpreted as being supportive of this “vascular depression” hypothesis [40]. Greater progression of WML volume contributed to a stronger decline in mental functioning in all patients except for patients with cerebrovascular disease. This finding is somewhat counterintuitive but may be explained by our finding of little improvement in physical functioning in patients with cerebrovascular disease. It could be that as a result of the substantial impairments and disability already associated with stroke lesions, increased progression of WML volume does not substantially contribute to the decline in mental functioning in these patients.

## 5. Conclusion

In summary, in patients with different manifestations of atherosclerotic disease, we found that physical functioning was mainly impaired in the acute phase after a symptomatic atherosclerotic event and improved during four years of followup, although improvement in physical functioning remained substantially lower in patients with cerebrovascular disease. Mental functioning was relatively unimpaired in the early phase, but declined in the four years thereafter. Greater progression of WML volume contributed to an even stronger decline in mental functioning in patients with symptomatic atherosclerotic disease. Considering the substantial impact on well-being and previously reported increased risk of adverse events associated with lower mental and physical functioning, further research should investigate whether modification of WML through better control of vascular risk factors could influence the course of HRQoL in patients with symptomatic atherosclerotic disease.

##  Conflict of Interests

The authors report no conflict of interests.

## Figures and Tables

**Table 1:** Baseline characteristics of patients with complete data at followup and of those lost to followup.

	Complete data at followup (*n* = 486)	Lost to followup (*n* = 503)
Age^¥^ (years)	58 ± 9.3	60 ± 10.2
Male gender (%)	80	79
Diagnosis of symptomatic atherosclerotic disease^‡^		
(i) Peripheral arterial disease	19	26
(ii) Coronary artery disease	65	61
(iii) Cerebrovascular disease	24	23
(iv) Abdominal aortic aneurysm	5	11
Severe atherosclerotic disease^*£*^	11	9
Smoking^†^ (pack/years)	21 (0–53)	18 (0–50)
Alcohol use		
(i) Never	13	18
(ii) Former	7	11
(iii) Current	79	72
Hypertension (%)	50	57
Diabetes mellitus (%)	16	25
Total intracranial volume^¥^ (mL)	1467 ± 127	1457 ± 132
Absolute total WML volume^†^ (mL)	1.3 (0.4–5.8)	1.7 (0.6–8.3)
Physical functioning^†^	44 (29–55)	43 (26–54)
Mental functioning^†^	51 (32–60)	48 (29–60)

WML: white matter lesions; mRS: modified Rankin Scale.

^‡^ The different groups of symptomatic atherosclerotic disease do not add up to the total study sample of 486, because various locations of symptomatic atherosclerotic disease can occur within one patient.

^*£*^Defined as patients with coronary artery disease and three-vessel or left main disease at inclusion, patients with cerebrovascular disease and a mRS grade ≥2 at inclusion, or patients with peripheral arterial disease with Fontaine grade ≥3 at inclusion.

^¥^Mean ± SD

^†^ Median, (10th–90th percentile).

**Table 2:** Baseline characteristics.

	Total sample (*n* = 486)	Peripheral arterial disease (*n* = 90)^‡^	Coronary artery disease (*n* = 318)^‡^	Cerebrovascular disease (*n* = 115)^‡^	Abdominal aortic aneurysm (*n* = 26)^‡^
Age^¥^ (years)	58 ± 9.3	56 ± 10.2	58 ± 9.0	59 ± 9.9	62 ± 7.9
Male gender (%)	80	66	86	76	96
Smoking^†^ (pack/years)	21 (0–53)	26 (1–56)	18 (0–51)	22 (0–53)	32 (7–76)
Alcohol use					
(i) Never	13	16	12	15	8
(ii) Former	7	10	8	4	4
(iii) Current	79	74	80	82	89
Hypertension (%)	50	58	47	61	54
Diabetes mellitus (%)	16	17	16	18	27
Total intracranial volume^¥^ (mL)	1467 ± 127	1437 ± 132	1474 ± 123	1467 ± 128	1507 ± 125
Absolute total WML volume^†^ (mL)	1.3 (0.4–5.8)	1.4 (0.5–4.6)	1.3 (0.3–4.7)	2.2 (0.4–11.2)	1.8 (0.5–10.3)
Physical functioning^†^	44 (29–55)	40 (20–53)	44 (31–55)	46 (31–56)	43 (32–55)
Mental functioning^†^	51 (32–60)	50 (33–60)	51 (31–60)	51 (34–59)	52 (34–58)

WML: white matter lesions.

^‡^The different groups of symptomatic atherosclerotic disease do not add up to the total study sample of 486, because various locations of symptomatic atherosclerotic disease can occur within one patient.

^¥^Mean ± SD

^†^ Median, (10th–90th percentile).
